# Early combination disease-modifying antirheumatic drug therapy and tight disease control improve long-term radiologic outcome in patients with early rheumatoid arthritis: the 11-year results of the Finnish Rheumatoid Arthritis Combination Therapy trial

**DOI:** 10.1186/ar3060

**Published:** 2010-06-24

**Authors:** Vappu Rantalaiho, Markku Korpela, Leena Laasonen, Hannu Kautiainen, Salme Järvenpää, Pekka Hannonen, Marjatta Leirisalo-Repo, Harri Blåfield, Kari Puolakka, Anna Karjalainen, Timo Möttönen

**Affiliations:** 1 Department of Internal Medicine, Centre for Rheumatic Diseases, Tampere University Hospital, PO Box 2000, FI-33521 Tampere, Finland; 2 Helsinki Medical Imaging Center, University of Helsinki, Tukholmankatu 8B, PO Box 20, 00014 Helsinki, Finland; 3 Orton Foundation, Tenholantie 10, 00280 Helsinki, Finland; 4 Medcare Foundation, Hämeentie 1, 44100 Äänekoski, Finland; 5 Jyväskylä Central Hospital, Keskussairaalantie 19, 40620 Jyväskylä, Finland; 6 Helsinki University Central Hospital, Stenbäckinkatu 9, 00290 Helsinki, Finland; 7 Seinäjoki Central Hospital, Hanneksenrinne 7, 60220 Seinäjoki, Finland; 8 Lappeenranta Central Hospital, Valto Käkelän katu 1, 53130 Lappeenranta, Finland; 9 Oulu University Hospital, PO Box 22, 90221 Oulu, Finland; 10 Turku University Hospital, PO Box 52, 20521 Turku, Finland

## Abstract

**Introduction:**

Early treatment of rheumatoid arthritis (RA) has been shown to retard the development of joint damage for a period of up to 5 years. The aim of this study was to evaluate the radiologic progression beyond that time in patients with early RA initially treated with a combination of three disease-modifying antirheumatic drugs (DMARDs) or a single DMARD.

**Methods:**

A cohort of 199 patients with early active RA were initially randomized to receive treatment with a combination of methotrexate, sulfasalazine, and hydroxychloroquine with prednisolone (FIN-RACo), or treatment with a single DMARD (initially, sulfasalazine) with or without prednisolone (SINGLE). After 2 years, the drug-treatment strategy became unrestricted, but still targeted remission. The radiographs of hands and feet were analyzed by using the Larsen score at baseline, 2, 5, and 11 years, and the radiographs of large joints, at 11 years.

**Results:**

Sixty-five patients in the FIN-RACo and 65 in the SINGLE group had radiographs of hands and feet available at baseline and at 11 years. The mean change from baseline to 11 years in Larsen score was 17 (95% CI, 12 to 26) in the FIN-RACo group and 27 (95% CI, 22 to 33) in the SINGLE group (*P *= 0.037). In total, 87% (95% CI, 74 to 94) and 72% (95% CI, 58 to 84) of the patients in the FIN-RACo and the SINGLE treatment arms, respectively, had no erosive changes in large joints at 11 years.

**Conclusions:**

Targeting to remission with tight clinical controls results in low radiologic progression in most RA patients. Patients treated initially with a combination of DMARDs have less long-term radiologic damage than do those treated initially with DMARD monotherapy.

****Trial registration**:**

Current Controlled Trials ISRCTN18445519.

## Introduction

Conservatively treated cohorts of rheumatoid arthritis (RA) patients have shown a constant deterioration of joint integrity [1,2]. However, treatment with traditional disease-modifying antirheumatic drugs (DMARDs) alone or in combinations [3,4] with glucocorticoids [5] as well as with biologic agents [6-9] has been shown to retard the progression of joint damage. Early therapy with combinations of conventional DMARDs has been shown to retard the radiologic progression of RA for a period of up to 5 years [4,10], but the effects of initial aggressive DMARD therapy on radiologic prognosis after that are unknown.

We previously demonstrated that early RA patients treated with a combination of DMARDs (methotrexate, sulfasalazine, and hydroxychloroquine with prednisolone) reached, at 2 years, more often clinical remission [3] and had less radiographic progression at 2 years [3] and at 5 years [10] than did patients initially treated with a single DMARD. We also reported that, at 11 years, most patients in both treatment groups had low disease activity and well-preserved function, but the combination DMARD-group patients reached remission more often than did those treated initially with a single DMARD [11].

In this study, we explored the effects of initial treatment strategy on the long-term radiographic findings at 11 years.

## Materials and methods

### Patients

From April 1993 to May 1995, 199 DMARD-naïve patients with recent-onset RA were admitted to this randomized study comparing the efficacy and tolerability of treatment with either a combination of DMARDs (starting with methotrexate, sulfasalazine, and hydroxychloroquine with prednisolone; FIN-RACo strategy) or a single DMARD (initially sulfasalazine with or without prednisolone; SINGLE strategy). The treatment was targeted toward remission in all patients. After 2 years, the treatment of RA was unrestricted, but still aiming at remission. Thus, regardless of the original randomization group, the patients could be treated liberally with DMARDs, biologic agents, glucocorticoids, and with their combinations, as clinically indicated and tolerated. Conversely, in long-term remission the protocol required drug doses to be reduced and eventually tapered off. The patient-selection criteria and the study design were described in detail earlier [3,10,11].

#### Radiologic assessment

Hands and feet of all patients were radiographed at baseline and at 2, 5, and 11 years. Hip, knee, elbow, and shoulder joints of the patients were radiographed at 11 years in 13 study centers; in two study centers, only clinically symptomatic large joints were radiographed. Total joint replacements were counted from the radiographs as well as from the patients' medical records. The radiographs were assessed by the same experienced radiologist (LL), who was blinded to the clinical data but aware of the order of the radiographs. The radiographs of hands and feet were scored according to the method of Larsen *et al. *[12], with a range from 0 to 200. The large joints were also scored according to the method of Larsen [12], and a score of ≥2 was considered to indicate erosive disease.

Clinical assessments were performed by the treating rheumatologist. DMARD strategies used between years 2 and 11 were carefully elucidated based on the patient's self-report and his or her medical records [11].

### Ethical considerations

The study was performed according to the principles of the Declaration of Helsinki. The protocol was approved by the national health authorities and ethics committees in all 18 participating hospitals. All patients gave written informed consent.

### Statistical methods

The data are presented as means with standard deviations (SDs), medians with interquartile ranges (IQRs), or counts with percentages. Statistical comparison between groups was made by *t *test, permutation test, χ^2 ^test, or the Fisher Exact test, when appropriate. The 95% confidence intervals (95% CIs) for the Larsen score are obtained by bias-corrected bootstrapping due to the skewed distribution. The difference in crude changes in Larsen score between the groups was tested by a permutation test. A random coefficient model with bootstrapped standard errors was adapted to analyze the progression of the Larsen score during 11 years and to compare the groups in time. An ordered logistic regression analysis was used to estimate the prediction of achieving radiologic progression. The adjusted risk ratio (RR) between the groups for having no erosive changes in large joints was estimated by a generalized linear model (log link), with presence of erosion in hands or feet at baseline as covariate. A time-to-event analysis based on the product-limit estimate of the cumulative "survival" function (Kaplan-Meier) was used to describe the time to the first total joint replacement. A log-rank test was used to identify any survival difference between the groups.

## Results

Of the 199 patients originally randomized to the study, 195 started treatment, 97 in the FIN-RACo group, and 98, in the SINGLE group. At the 11-year visit, 68 patients were assessed in the FIN-RACo group, and 70, in the SINGLE group; the patients' baseline demographic and clinical characteristics were comparable [11]. In total, 130 patients had radiographs of hands and feet available at baseline and at 11 years, 65 cases in each group.

A trend toward a higher mean (range) Larsen score at baseline was found in the SINGLE group compared with the FIN-RACo group: 5 (0 to 30) versus 3 (0 to 25) (*P *= 0.069). Furthermore, the dropout cases in the FIN-RACo group had a higher mean ± SD Larsen score at baseline than did the completers: 6 ± 9 versus 3 ± 6 (*P *= 0.037). In the SINGLE group, the baseline Larsen scores did not differ between the dropouts and the completers: 3 ± 5 versus 5 ± 7 (*P *= 0.22).

**Figure 1:**
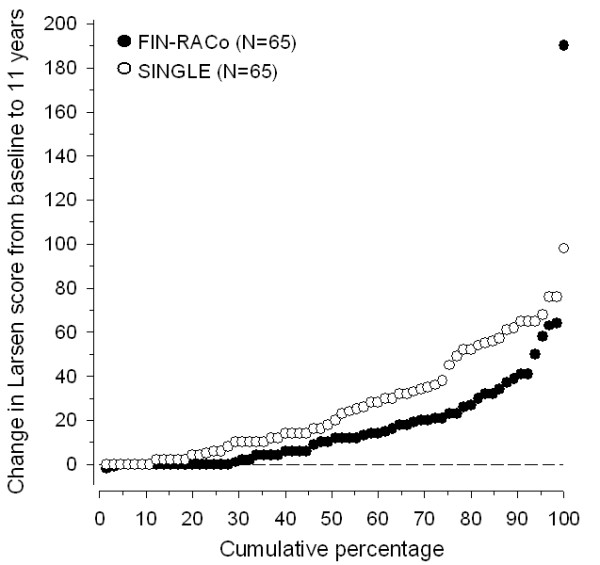
**The cumulative percentage of increase in Larsen score from baseline to 11 years in patients initially randomized to receive a combination (FIN-RACo) of disease-modifying antirheumatic drugs (DMARDs) or a single DMARD (SINGLE)**.

The cumulative percentages of Larsen scores in both groups are shown in Figure 1. One outlier in the FIN-RACo group had progressed to almost a maximum score after 11 years. Despite active combination DMARD treatment, this patient had had high disease activity and HAQ score throughout the follow-up, and by 11 years, also had damage in large joints as well as one total joint replacement.

**Figure 2:**
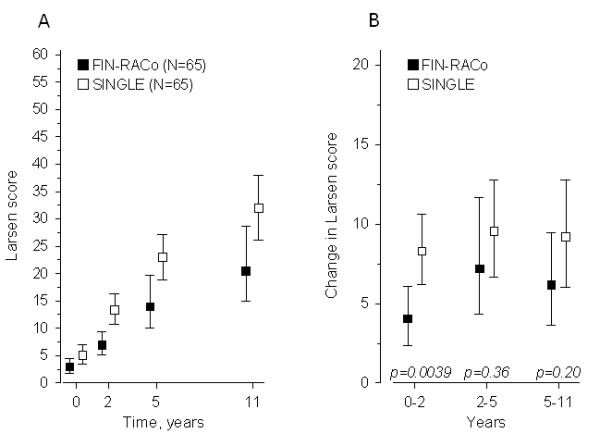
**The crude mean Larsen scores of hands and feet at baseline and at 2, 5, and 11 years in patients initially randomized to receive a combination (FIN-RACo) of disease-modifying antirheumatic drugs (DMARDs) or a single DMARD (SINGLE)**. **(a) **Included are the subjects scored at 11 years (two patients in each group did not have scores at 5 years). Values are expressed as the mean and 95% confidence interval. **(b) **The mean changes in Larsen score during years 0 to 2, 2 to 5, and 5 to 11, according to the initial treatment groups.

The mean Larsen scores of hands and feet at baseline and at 2, 5, and 11 years in both groups are shown in Figure 2. The crude mean change from baseline to 11 years in Larsen score was 17 (95% CI, 12 to 26) in the FIN-RACo group and 27 (95% CI, 22 to 33) in the SINGLE group (*P *= 0.037). When using all time points (0, 2, 5, and 11 years) and adjusting for Larsen score at baseline, the progression of Larsen score differed statistically significantly between the groups (*P *= 0.021, for Time-by-Group interaction effect), with the FIN-RACo group having on average lower progression (*P *< 0.001, for Group-Effect) (Figure 2a). In an ordered logistic regression analysis, the extent of joint-damage progression in hands and feet at 11 years was predicted by the presence of serum rheumatoid factor at baseline and by the single-treatment strategy for the first 2 years (Table 1).

**Table 1:** Ordered logistic regression analysis for radiologic progression at 11 years

Variable at baseline	Odds ratio (95% CI)	*P *value
Female sex	1.74 (0.84 to 3.60)	0.13
Age, years	0.99 (0.96 to 1.02)	0.60
Disease duration before diagnosis, months	1.02 (0.94 to 1.10)	0.68
Rheumatoid factor positivity	3.17 (1.45 to 6.92)	0.004
Erythrocyte sedimentation rate	1.01 (0.99 to 1.02)	0.33
Larsen score	0.99 (0.94 to 1.05)	0.77
Initial randomization group		0.016
FIN-RACo	1.00 (reference)	
SINGLE	2.39 (1.78 to 4.84)	

Radiologic progression in hands and feet was determined according to the tertiles of Larsen score changes (categories 0 to 1, 2 to 17, and ≥18). FIN-RACo, study group treated for the first 2 years with a combination of three disease-modifying antirheumatic drugs, initially methotrexate, sulfasalazine, and hydroxychloroquine, with prednisolone; SINGLE, study group treated for the first 2 years with one disease-modifying antirheumatic drug, initially sulfasalazine, with or without prednisolone.

**Figure 3:**
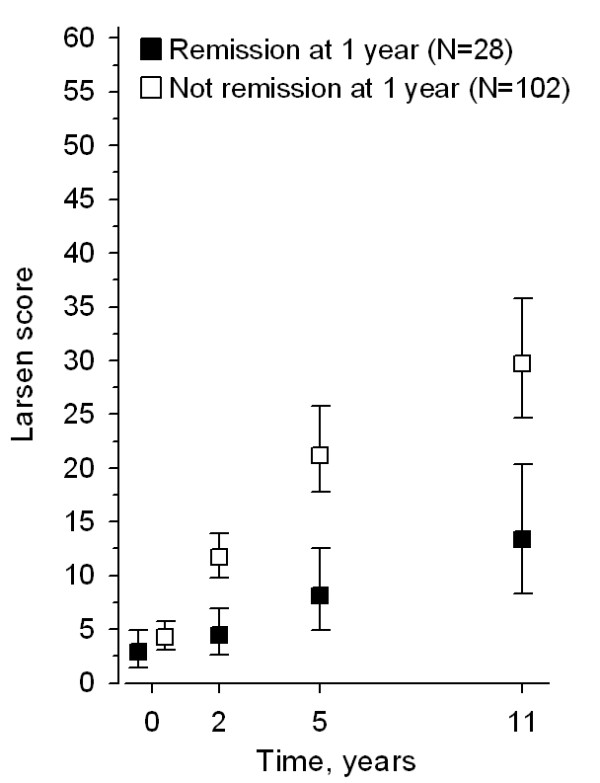
**The crude mean Larsen scores of hands and feet at baseline, and at 2, 5, and 11 years in patients who had been in remission at 1 year and in patients who had not been in remission at 1 year**. Included are the subjects scored at 11 years (two patients in each group did not have scores at 5 years). Values are expressed as the mean and 95% confidence interval.

The crude mean change from baseline to 11 years in Larsen score was 10 (95% CI, 6 to 16) in patients who had been in remission at 1 year and 25 (95% CI, 21 to 31) in patients who had not been in remission at 1 year (*P *= 0.001). When using all time points (0, 2, 5, and 11 years) and adjusting for Larsen score at baseline, the progression of Larsen score differed statistically significantly between the patients in remission and not in remission at 1 year (*P *< 0.001, for Time-by-Group interaction effect), with the patients in remission at 1 year having on average lower progression (*P *< 0.001, for Group-Effect) (Figure 3).

At 11 years, 52 and 54 patients in FIN-RACo and in SINGLE groups, respectively, had all the large joints radiographed. In FIN-RACo and SINGLE groups, 87% (95% CI, 74 to 94) and 72% (95% CI, 58 to 84), respectively, of these patients had no erosive changes in large joints at 11 years (RR, 1.22 (95% CI, 0.99 to 1.50)). The number of damaged large joints (Larsen score, ≥2) did not differ between the groups (Table 2).

**Table 2:** Number (percentage) of RA patients with damage to any or to multiple large joints as well as with uni- or bilateral erosive (Larsen score ≥2) large joints after 11 years of follow-up, by initial randomization group

	Original randomization group			
	**FIN-RACo (n = 52)**		**SINGLE (n = 54)**	

Damage to any large joint	7 (13%)		15 (28%)	
Damage to multiple (two to three) large joints	5 (10%)		10 (19%)	

**Radiographed joint**	**Unilateral damage**	**Bilateral damage**	**Unilateral damage**	**Bilateral damage**

Shoulder	0	2 (4%)	4 (7%)	7 (13%)
Elbow	1 (2%)	0	1 (2%)	1 (2%)
Hip	3 (6%)	2 (4%)	4 (7%)	1 (2%)
Knee	3 (6%)	1 (2%)	2 (4%)	0

FIN-RACo, study group treated for the first 2 years with a combination of three disease-modifying antirheumatic drugs, initially methotrexate, sulfasalazine, and hydroxychloroquine, with prednisolone; RA, rheumatoid arthritis; SINGLE, study group treated for the first 2 years with one disease-modifying antirheumatic drug, initially sulfasalazine, with or without prednisolone.

Nine patients (four in the FIN-RACo and five in the SINGLE group) had altogether 12 total joint replacements (six knees and six hips). Of these, two arthroplasties had been performed because of primary osteoarthrosis of the knee, and one, because of hip fracture. The occurrence of total joint replacements did not differ between the FIN-RACo and the SINGLE treatment groups: 6% (95% CI, 2 to 16) versus 8% (95% CI, 3 to 18) (*P *= 0.73) during the follow-up.

**Figure 4:**
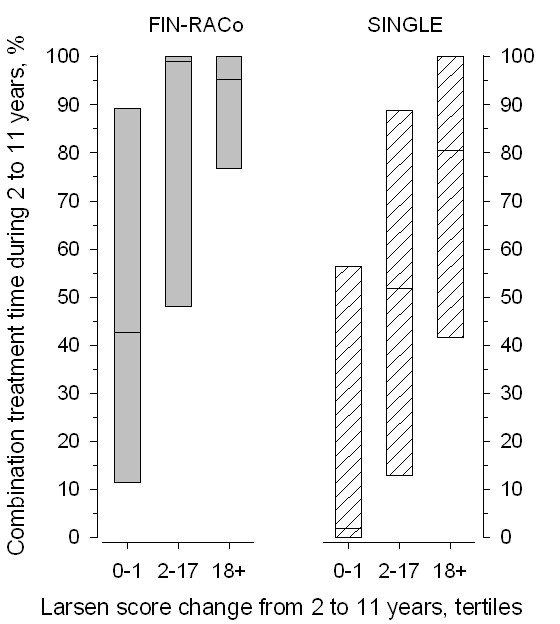
**Percentage of treatment time using combination DMARD strategy between year 2 and year 11 in patients of the original randomization groups divided into tertiles, according to change in Larsen score of hands and feet from year 2 to year 11**. Values are expressed as median and interquartile range.

Treatment strategies used between 2 to 11 years were reported previously [11]. In both groups, the patients in the tertile of the lowest radiologic progression in hands and feet from year 2 to year 11 (change in Larsen score, 0 to 1) had received significantly shorter periods of combination-DMARD treatments between years 2 and 11 than did the patients with intermediate (change in Larsen score, 2 to 17) or high (change in Larsen score, ≥18) progression rates (*P *= 0.001 for linearity in both treatment groups) (Figure 4). A similar trend was found for biologic treatments in the entire study population; 14 patients (11%) had received TNF-inhibitors; of these, one had low; five, intermediate; and eight, high radiographic progression between years 2 and 11.

## Discussion

The main finding of the present study is that targeting to remission with traditional DMARDs and tight clinical controls results in low radiologic progression in most RA patients. Still, patients treated initially with the FIN-RACo strategy during the first 2 years have less radiographic damage in small joints, even in long term than did those treated initially with DMARD monotherapy.

Less radiographic damage is found in RA patients at present than during previous decades [13]. In our study, both treatment arms had excellent radiologic small-joint outcome compared with historic cohorts. In a previous Finnish cohort of 103 patients with early RA, beginning in the 1970 s, the radiologic progression was steepest during the first 8 years but continued throughout the follow-up of 20 years [1]. In that cohort, the mean ± SD Larsen score at 3 years was 27 ± 21, and at 15 years, 78 ± 49. Thus, after 3 years of RA, the historic patients had comparable amounts of radiographic damage to the patients of the present study at 11 years. In a Swedish cohort starting in 1985, 181 patients with conservatively treated early RA had, at 10 years, a median Larsen score of 54 (IQR, 28 to 80) [2], thus double the Larsen score of our patients at 11 years. These findings are in accordance with those of Finckh *et al. *[13], who found that the radiographic prognosis of RA has improved during the past decades parallel to more active treatments.

Even though most patients had excellent radiographic results at 11 years, the patients treated with the FIN-RACo strategy had significantly lower increases in the median Larsen score from baseline to 11 years than did the SINGLE patients, and besides the presence of rheumatoid factor, only the initial SINGLE treatment predicted the radiographic progression at 11 years in the ordered logistic regression analysis. The main difference between the groups had developed during the first 2 years; after that, both groups progressed similarly. For unknown reasons, the dropout patients in the FIN-RACo group had a higher Larsen score at baseline than did those cases who completed the study. Thus, in the completers of the FIN-RACo group, a trend toward a lower Larsen score at baseline was seen compared with the SINGLE group completers. At worst, this fact may bias the study. However, in the statistical analysis adjusted with baseline Larsen score, a highly significant difference in radiologic progression was found between the groups. Therefore, we find it justified to conclude that the observed difference between the groups represents rather the results of a more-effective initial DMARD treatment strategy than a biologic bias.

For evaluating the radiographic damage, we used the Larsen score, which has been found to be less sensitive to change than the Sharp/van der Heijde method [14,15]. Conversely, the Larsen method tends to be more specific than the Sharp/van der Heijde method [14], and when the follow up is as long as 11 years, we prefer specificity over sensitivity; it is more important to distinguish clinically relevant from unspecific changes than to find subtle joint-space narrowing. Also, the intraobserver reliability in Larsen score is somewhat better than that of the Sharp/van der Heijde method [15,16], and because we have had the same experienced radiologist scoring the radiographs with the Larsen method throughout the follow-up, we find this method logical. To our knowledge, no other methods exist for evaluating the radiographic progression in large joints besides the Larsen method.

Only 13% of the FIN-RACo and 28% of the SINGLE patients had some radiographic damage in large joints. Few long-term studies of early RA assess large-joint damage, and none of them have a definite treatment protocol. One study, published in 1997, found radiographic damage in large joints in 50% of the patients after 6 years of RA [17]. In a Dutch study, 54% of patients had at least one eroded large joint after 12 years of RA [18]. In the present study, the infrequent destruction of large joints was also reflected in the small number of total joint replacements in both of our treatment groups compared with earlier cohorts [19], even though the follow-up of 11 years is too short to evaluate the final incidence of total joint replacements.

Probably the most important precondition to our excellent results in most patients was the active treatment policy aiming at remission at all time points. Even though recent reports showed that radiologic progression may occur even while the patient appears to be in remission [20], most damage still emerges in clinically inflamed joints [21]. Our results emphasize the importance of early remission for the long-term outcome of the patients. In the present study, the patients who had been in strict remission at 1 year had significantly less radiologic progression throughout the follow-up than did the patients who had not reached remission at 1 year. Remissions were reached more often by the FIN-RACo arm patients than by the SINGLE patients at 2 years [3], as well as at 11 years [11], but patients in both treatment arms had mainly low disease activity and well-preserved function throughout the follow-up [11]. This clinical profile fits the radiologic profile of our study groups well; compared with less aggressively treated patients, both groups were doing well, but the FIN-RACo patients even better.

We earlier reported that during the liberal treatment phase between years 2 and 11, the use of DMARDs differed between groups, with combination treatments used more often in the original FIN-RACo group [11]. This difference had, however, no impact on the clinical outcome at 11 years. In the FIN-RACo group, the patients who had low disease activity at 11 years had received significantly shorter periods of combination DMARDs between 2 and 11 years than had the patients who had high disease activity at 11 years [11]. Similarly, in the present study, the patients with the least radiologic progression after year 2 had received the shortest periods of combination DMARD strategy after 2 years. These results are in agreement with the fact that in longitudinal observational studies, the cases treated most intensively are the most likely ones to have the most severe disease [22]. And yet, aggressive treatments in established disease do not seem to gain as much effect as they do in early disease. This emphasizes the importance of early, effective treatment and tight control of therapeutic response. Late strengthening of DMARD treatment is not able to reverse the damage already arisen. Nevertheless, it is probable that radiologic progression would have been even steeper had the treatments during the liberal phase been less aggressive.

Glucocorticoids were a part of the FIN-RACo strategy and were allowed in the SINGLE strategy to reach remission. Because glucocorticoids have been shown to retard radiologic progression [5], it could be hypothesized that their use would explain the difference in Larsen score between the groups. However, the patients treated with the FIN-RACo strategy needed fewer intraarticular glucocorticoid injections and had a smaller cumulative dose of glucocorticoids during the first 2 years than did the SINGLE strategy group [3]. Thus, the better radiologic outcome in the FIN-RACo arm does not seem to depend on the use of glucocorticoids, but rather on the more effective and rapidly working DMARDs during the critical "window of opportunity." Whether the difference between the groups would have been smaller, had the first DMARD in the SINGLE strategy been methotrexate, cannot be answered by this study. However, the SINGLE strategy was not tied to sulfasalazine but to a strategy of using one DMARD at a time, and, during the first 2 years, 52% of patients in the SINGLE group were switched to methotrexate [3].

## Conclusions

We conclude that treating RA from the very beginning actively and aggressively with DMARDs, including tight clinical control and aiming for remission, pays off, even in the long run. Further, the patients treated initially with the FIN-RACo strategy manage better than the cases treated actively with the SINGLE strategy. Both small and large peripheral joints are spared. Consequently, the need for joint-replacement operations decreases. Clinical disease activity remains low, functional capacity well preserved, and life expectancy normal [11]. Further studies will reveal whether all this is reflected in the maintenance of working capacity.

## Abbreviations

CI: confidence interval; DMARD: disease-modifying antirheumatic drug; FIN-RACo: study group treated for the first 2 years with a combination of three disease-modifying antirheumatic drugs: initially methotrexate: sulfasalazine: and hydroxychloroquine: with prednisolone; HAQ: health assessment questionnaire; IQR: interquartile range; RA: rheumatoid arthritis; RR: risk ratio; SD: standard deviation; SINGLE: study group treated for the first 2 years with one disease-modifying antirheumatic drug: initially sulfasalazine: with or without prednisolone; TNF: tumor necrosis factor.

## Competing interests

The authors declare that they have no competing interests.

## Authors' contributions

VR participated in the acquisition of data, performed the statistical analysis with HK and SJ, and drafted the manuscript. MK, PH, ML-R, and TM belong to the advisory board of the FIN-RACo study, which is responsible for the study design; they also participated in the acquisition of data and helped to draft the manuscript. LL scored the patients' radiographs and participated in drafting the manuscript. HB, KP, and AK participated in the acquisition of data and helped to draft the manuscript. All authors have been involved in drafting the manuscript and have given final approval of the version to be published.
